# A New One-Pot Synthesis of Quinoline-2-carboxylates under Heterogeneous Conditions

**DOI:** 10.3390/molecules21060776

**Published:** 2016-06-15

**Authors:** Serena Gabrielli, Alessandra Giardinieri, Susanna Sampaolesi, Roberto Ballini, Alessandro Palmieri

**Affiliations:** Green Chemistry Group, Chemistry Division, School of Science and Technology, University of Camerino, via S. Agostino 1, 62032 Camerino, Italy; serena.gabrielli@unicam.it (S.G.); alessand.giardinieri@studenti.unicam.it (A.G.); susanna.sampaolesi@unicam.it (S.S.); roberto.ballini@unicam.it (R.B.)

**Keywords:** quinolines, β-nitroacrylates, one-pot processes, solid supported reagents, heterocycles

## Abstract

Quinoline-2-carboxylates are an important subclass of quinoline derivatives largely present in a variety of biologically active molecules, as well as useful ligands in metal-catalyzed reactions. Herein, we present a new one-pot protocol for synthesizing this class of derivatives starting from β-nitroacrylates and 2-aminobenzaldehydes. In order to optimize the protocol, we investigated several reaction conditions, obtaining the best results using the 2-*tert*-Butylimino-2-diethylamino-1,3-dimethylperhydro-1,3,2-diazaphosphorine (BEMP) as solid base, in acetonitrile. Finally, we demonstrated the generality of our approach over several substrates which led to synthesize a plethora of functionalized quinolines-2-carboxylate derivatives in good overall yields.

## 1. Introduction

**Scheme 1:**
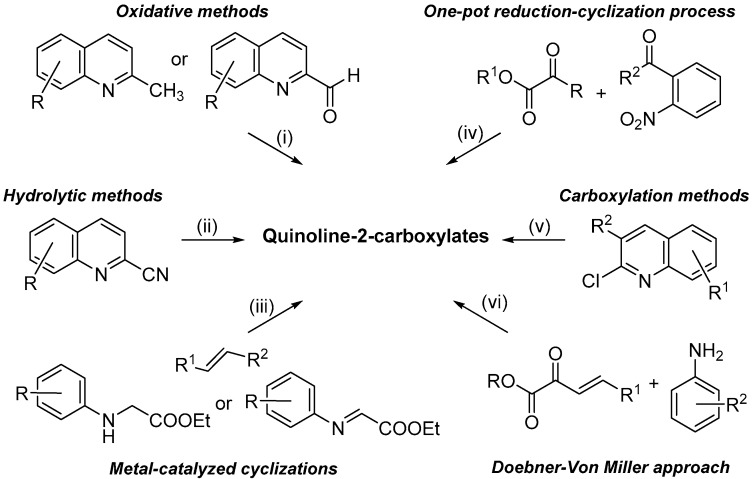
Main synthetic approaches for synthesizing quinoline-2-carboxylate derivatives.

Quinoline constitutes one of the most important nitrogen-containing heterocyclic compounds possessing several biological activities such as *anti*-bacterial, *anti*-fungal, *anti*-malarial, *anti*-convulsant, *anti*-inflammatory, anthelmintic, cardiotonic and analgesic [1]. In addition, the quinolinic nucleus is largely occurring in natural compounds and deeply investigated as building blocks for synthesizing highly functionalized materials [2,3]. In particular, quinoline-2-carboxylates play a predominant role as precursor of biologically active molecules [4,5,6,7,8] and as useful ligands in metal-catalyzed reactions [9,10]. Due to their importance, over the years, a variety of synthetic methodologies have been reported in the literature, which can be classified (Scheme 1) in: (i) oxidative processes, starting from the corresponding 2-methyl or 2-carbonyl quinolines [11,12]; (ii) hydrolytic reactions of cyano precursors [13]; (iii) metal-catalyzed cyclization, starting from *N*-aryl glycine derivatives or iminoethyl glyoxylates [14,15]; (iv) one-pot reduction-cyclization processes of 2-acylnitroarenes with α-oxoesters [16]; (v) carboxylation reactions of 2-chloro quinoline derivatives [17]; and (vi) the historical Doebner-Von Miller protocol [18].

**Scheme 2:**
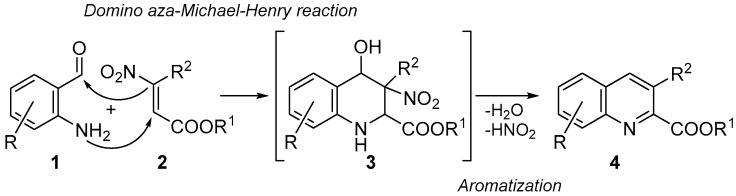
Synthetic approach for synthesizing quinoline-2-carboxylate derivatives **4**.

Nevertheless, several of the reported procedures present some limitations such as restricted applicability (only few examples were reported) [11,12,13,18], low overall yields [16] and harsh reaction conditions [17]. Hence, new simple and general protocols for synthesizing these derivatives would surely be welcome. In this context, following our studies on the chemistry of β-nitroacrylates as valuable precursors of heterocycle systems [19,20,21,22,23,24,25,26,27], we propose their application as strategic starting materials, in combination with 2-aminobenzaldheydes, for synthesizing quinoline-2-carboxylates. The idea was to develop a new one-pot process involving four different reactions: (i) an *aza*-Michael addition between the 2-aminobenzaldheydes **1** and β-nitroacrylates **2**; followed by (ii) an intramolecular Henry reaction to give the benzopiperidines **3**; (iii) elimination of water; and (iv) nitrous acid elimination to provide the aromatization of the piperidine core, and thus the formation of title targets **4** (Scheme 2).

## 2. Results and Discussion

**Scheme 3:**
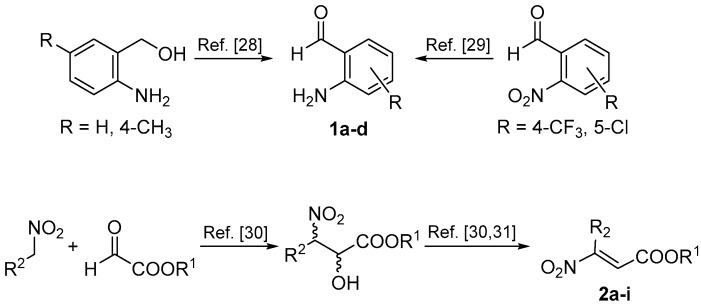
Synthesis of starting materials **1** and **2**.

In order to study the reaction, we first synthesized the starting materials (Scheme 3). 2-Aminobenzaldheydes were prepared from the corresponding alcohols or nitro precursors [28,29], while β-nitroacrylates were synthesized by the Henry reaction-elimination process, starting from nitroalkanes and alkyl glyoxalates [30,31].

Once the starting materials were prepared, we switched our attention to optimize the process. Thus, first we studied the “*aza*-Michael-Henry domino process” between 2-aminobenzaldheyde **1a** and ethyl-3-nitropent-2-enoate **2a** (Table 1). Based on our previous experiences, we started testing the reaction under promoter-free and solvent-free conditions and, after a series of trials, the best result was obtained using a slight excess of **2a** (1.1 equivalent) at 70 °C (**3aa**, 66%, entry 7, the presence of solvents or bases makes the reaction unproductive entry 9–12).

Successively, we studied the conversion of **3aa** into **4aa** (Table 2) and, after deep screening in terms of bases, solvents and temperature, we found the best yield of **4aa** (86%, entry 8) at 50 °C, in acetonitrile using 1.25 equivalents of BEMP on polymer [32].

**Scheme 4:**
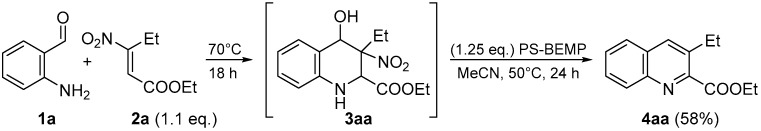
One-pot process.

At that point, in order to achieve a one-pot process, we combined the two steps obtaining the quinoline **4aa** in 58% of overall yield (Scheme 4).

**Scheme 5:**
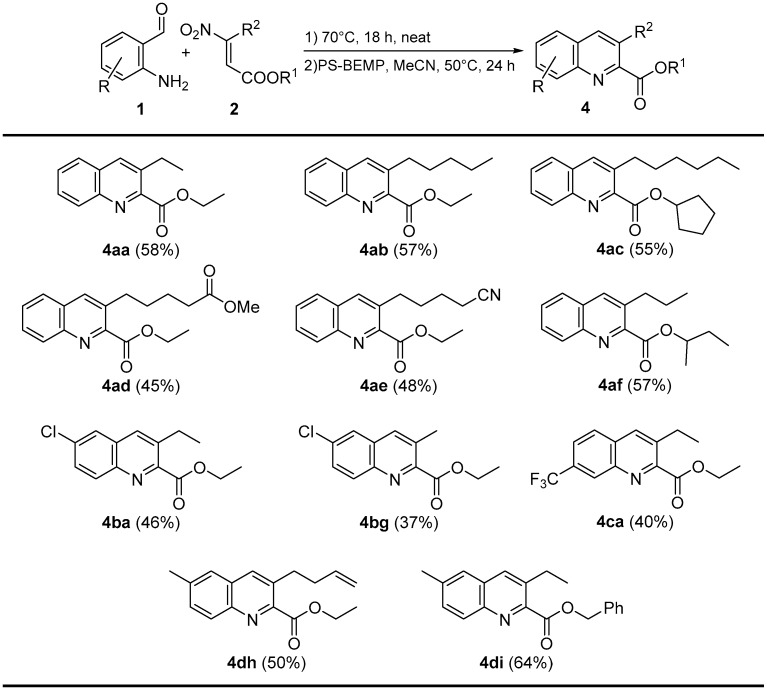
One-pot reaction products.

Finally, with the aim to demonstrate the generality of our method, we tested our reaction conditions on a wide range of 2-aminobenzaldheydes **1** and β-nitroacrylates **2**. In all cases, the products were obtained in moderate to good overall yields (37%–64%), even in the presence of a variety of functionalities (Scheme 5).

## 3. Conclusions

In conclusion, we developed a new general and valuable one-pot procedure for synthesizing an important class of quinolines such as quinoline-2-carboxylates. In our approach, it was possible to prepare title compounds in good overall yields, introducing different substituent in 3-position as well as in the benzene ring. Furthermore, the mildness of our reaction conditions allowed preserving several functionalities such as ester, cyano, chlorine, fluorine and carbon–carbon double bond. In addition, the use of supported BEMP enabled us to minimize the use of materials, avoiding any complex aqueous work-up, with evident advantages from a sustainable point of view. Finally, we still demonstrated the usefulness of β-nitroacrylates as a valuable precursor of heterocyclic systems.

## 4. Experimental Section

### 4.1. General Section

^1^H-NMR were recorded at 400 MHz on a VarianMercury Plus 400. ^13^C-NMR were recorded at 100 MHz. IR spectra were recorded with a PerkineElmer Paragon 500 FT-IR. Mass spectra were performed on a GC/MS system by means of the EI technique (70 eV). Microanalyses were performed with a CHNS-O analyzer Model EA 1108 from Fisons Instruments.

### 4.2. Chemistry Section

General Procedure for the Preparation of Compounds **4**

A mixture of 2-aminobenzaldheydes **1** (1 mmol) and β-nitroacrylates **2** (1.1 mmol) was stirred, under solvent-free conditions, at 70 °C for 18 h. Then, after that the temperature was diminished at 50 °C, acetonitrile (10 mL) and PS-BEMP (1.25 mmol, 0.570 mg) were added, and the resulting solution was stirred at 50 °C for further 24 h. Finally, the resin was filtered off washing with fresh EtOAc (10 mL) and the crude products **4**, obtained after removal of the solvent at reduced pressure, were purified by flash chromatography column (hexane–ethyl acetate 9:1).

*Compound* **4aa**. Pale yellow oil. IR (cm^−1^, neat): 1063, 1160, 1458, 1619, 1727, 2959. ^1^H-NMR (CDCl_3_, 400 MHz) δ: 1.34 (t, 3H, *J* = 7.7 Hz), 1.46 (t, 3H, *J* = 7.3 Hz), 3.01 (q, 2H, *J* = 7.7 Hz), 4.53 (q, 2H, *J* = 7.3 Hz), 7.53–7.60 (m, 1H), 7.65–7.72 (m, 1H), 7.78 (d, 1H, *J* = 8.1 Hz), 8.04 (s, 1H), 8.16 (d, 1H, *J* = 8.5 Hz). ^13^C-NMR (CDCl_3_, 100 MHz) δ: 14.5, 15.4, 25.9, 62.2, 127.2, 128.2, 129.1, 129.5, 130.0, 135.6, 136.4, 146.0, 150.7, 167.1. GC-MS (70 eV): *m*/*z*: 229 ([M^+^], 29), 200 (83), 182 (14), 157 (39), 156 (99), 155 (63), 154 (100), 128 (34). Anal. Calcd. for C_14_H_15_NO_2_ (229.28): C, 73.34; H, 6.59; N, 6.11. Found: C, 73.39; H, 6.62; N, 6.08.

*Compound* **4ab**. Pale yellow oil. IR (cm^−1^, neat): 1073, 1171, 1459, 1610, 1728, 2952. ^1^H-NMR (CDCl_3_, 400 MHz) δ: 0.9 (t, 3H, *J* = 7.3 Hz), 1.34–1.41 (m, 4H), 1.47 (t, 3H, *J* = 7.3 Hz), 1.65–1.74 (m, 2H), 2.96–2.99 (m, 2H), 4.53 (q, 2H, *J* = Hz 7.3), 7.55–7.60 (m, 1H), 7.67–7.72 (m, 1H), 7.79 (d, 1H, *J* = 8.1 Hz), 8.04 (s, 1H), 8.175 (d, 1H *J* = 8.5 Hz). ^13^C-NMR (CDCl_3_, 100 MHz) δ: 14.1, 14.4, 22.6, 30.1, 31.8, 32.7, 62.1, 127.1, 128.1, 129.0, 129.4, 130, 134.3, 137.1, 146.0, 151.0, 167.1. GC-MS (70 eV): *m*/*z*: 271 ([M^+^], 25), 245 (68), 228 (72), 224 (44), 199 (14), 198 (96), 196 (26), 182 (35), 168 (38), 166 (29), 156 (22), 155 (20), 154 (100), 143 (72), 142 (43), 141 (15), 140 (19), 132 (14), 115 (38). Anal. Calcd. for C_17_H_21_NO_2_ (271.36): C, 75.25; H, 7.80; N, 5.16. Found: C, 75.29; H, 7.77; N, 5.13.

*Compound* **4ac**. Pale yellow oil. IR (cm^−1^, neat): 1069, 1167, 1455, 1615, 1722, 2955. ^1^H-NMR (CDCl_3_, 400 MHz) δ: 0.84–0.92 (m, 3H), 1.23–1.44 (m, 6H), 1.59–1.73 (m, 4H), 1.74–1.86 (m, 2H), 1.89–2.11 (m, 4H), 2.88–2.94 (m, 2H), 5.51–5.58 (m, 1H), 7.53–7.58 (m, 1H), 7.71–7.75 (m, 1H), 7.77 (d, 1H, *J* = 8.1 Hz), 8.02 (s, 1H), 8.17 (d, 1H, *J* = 8.5 Hz). ^13^C-NMR (CDCl_3_, 100 MHz) δ: 14.3, 22.8, 24.1, 29.5, 31.4, 31.9, 32.8, 32.9, 79.4, 127.2, 128.1, 128.9, 129.6, 129.8, 133.7, 137.2, 145.8, 151.5, 167.2. GC-MS (70 eV): *m*/*z*: 325 ([M^+^], 4), 257(14), 256 (73), 238 (14), 213 (18), 212 (100), 200 (20), 182 (17), 168 (19), 157 (16), 155 (26), 143 (55), 142 (61), 115(20). Anal. Calcd. for C_21_H_27_NO_2_ (325.45): C, 77.50; H, 8.36; N, 4.30. Found: C, 77.55; H, 8.39; N, 4.27.

*Compound* **4ad**. Pale yellow oil. IR (cm^−1^, neat): 1074, 1179, 1436, 1615, 1730, 2949. ^1^H-NMR (CDCl_3_, 400 MHz) δ: 1.46 (t, 3H, *J* = 7.3 Hz), 1.70–1.76 (m, 4H), 2.31–2.39 (m, 2H), 2.91–3.02 (m, 2H), 3.65 (s, 3H), 4.50 (q, 2H, *J* = 7.3 Hz), 7.57 (t, 1H, *J* = 7.3 Hz), 7.68 (t, 1H, *J* = 7.3 Hz), 7.77 (d, 1H, *J* = 8.1 Hz), 8.02 (s, 1H), 8.15 (d, 1H, *J* = 8.5 Hz). ^13^C-NMR (CDCl_3_, 100 MHz) δ: 14.5, 24.9, 30.8, 32.6, 34.0, 51.8, 62.3, 127.2, 128.3, 129.0, 129.7, 130.0, 133.8, 137.4, 146.0, 150.5, 167.0, 174.1. GC-MS (70 eV): *m*/*z*: 315([M^+^], 30), 286 (68), 284 (29), 270 (18), 242 (28), 236 (21), 228 (81), 182 (61), 180 (29), 170 (14), 168 (42), 167 (44), 156 (14), 155(22), 154 (100), 143 (52), 142 (22), 127 (14), 115 (27). Anal. Calcd. for C_18_H_21_NO_4_ (315.37): C, 68.55; H, 6.71; N, 4.44. Found: C, 68.59; H, 6.68; N, 4.47.

*Compound* **4ae**. Pale yellow oil. IR (cm^−1^, neat): 1027, 1236, 1456, 1659, 1716, 2245, 2933. ^1^H-NMR (CDCl_3_, 400 MHz) δ: 1.47 (t, 3H, *J* = 7.3 Hz), 1.71–1.93 (m, 4H), 2.40 (t, 2H, *J* = 6.8 Hz), 3.03 (t, 2H, *J* = 7.7 Hz), 4.53 (q, 2H, *J* = 7.3 Hz), 7.59 (t, 1H, *J* = 6.8 Hz), 7.71 (t, 1H, *J* = 7.3 Hz), 7.79 (d, 1H, *J* = 8.12 Hz), 8.04 (s, 1H), 8.17 (d, 1H *J* = 8.1 Hz). ^13^C-NMR (CDCl_3_, 100 MHz) δ: 14.5, 17.3, 25.4, 30.4, 32.1, 62.4, 119.7, 127.2, 128.5, 129.0, 129.9, 130.0, 133.3, 137.5, 146.2, 150.0, 166.9. GC-MS (70 eV): *m*/*z*: 282 ([M^+^], 9), 253 (29), 228 (34), 209 (38), 209 (38), 182 (19), 170 (23), 168 (48), 154 (73), 143 (100), 142 (18), 140 (18), 115 (33), 91 (15). Anal. Calcd. for C_17_H_18_N_2_O_2_ (282.34): C, 72.32; H, 6.43; N, 9.92. Found: C, 72.36; H, 6.47; N, 9.89.

*Compound* **4af**. Pale yellow oil. IR (cm^−1^, neat): 1072, 1162, 1460, 1617, 1720, 2958. 1H-NMR (CDCl_3_, 400 MHz) δ: 0.89–1.03 (m, 6H), 1.46 (d, 3H, *J* = 6.4 Hz), 1.66–1.79 (m, 3H), 1.80–1.93 (m, 1H), 2.93 (dt, 2H, *J* = 7.7, 2.1 Hz), 5.19–5.31 (m, 1H), 7.58 (t, 1H, *J* = 8.1 Hz), 7.71 (t, 1H, *J* = 8.1 Hz), 7.79 (d, 1H, *J* = 8.1 Hz), 8.05 (s, 1H), 8.24 (d, 1H, *J* = 8.1 Hz). ^13^C-NMR (CDCl_3_, 100 MHz) δ: 10.2, 14.3, 19.8, 24.8, 29.1, 35.0, 74.8, 127.5, 128.2, 129.0, 129.8, 130.3, 133.6, 137.8, 146.0, 151.6, 167.1. GC-MS (70 eV): *m*/*z*: 271 ([M^+^], 8), 215(20), 214 (100), 200 (20), 198 (17), 182 (14), 170 (59), 168 (63), 154 (28), 143 (93), 143 (24), 115 (21), 41 (18), 29(19). Anal. Calcd. for C_17_H_21_NO_2_ (271.36): C, 75.25; H, 7.80; N, 5.16. Found: C, 75.21; H, 7.77; N, 5.19.

*Compound* **4ba**. Pale yellow oil. IR (cm^−1^, neat): 1070, 1177, 1479, 1616, 1723, 2975. ^1^H-NMR (CDCl_3_, 400 MHz) δ: 1.31 (t, 3H, *J* = 7.3 Hz), 1.45 (t, 3H, *J* = 7.3 Hz), 2.99 (q, 2H, *J* = 7.3 Hz), 4.51 (q, 2H, *J* = 7.3 Hz), 7.57–7.63 (m, 1H), 7.73-7.76 (m, 1H), 7.93 (s, 1H), 8.08 (d, 1H, *J* = 9.0 Hz). ^13^C-NMR (CDCl_3_, 100 MHz) δ: 14.5, 15.2, 25.9, 62.4, 125.9, 129.7, 130.6, 131.6, 134.0, 135.5, 136.7, 144.3, 150.9, 166.8. GC-MS (70 eV): *m*/*z*: 263 ([M^+^], 26), 236 (25), 234 (71), 218 (15), 216 (16), 192 (30), 191 (54), 190 (100), 189 (59), 188 (57), 163 (19), 154 (52), 153 (14), 128 (14), 126 (18). Anal. Calcd. for C_14_H_14_ClNO_2_ (263.72): C, 63.76; H, 5.35; N, 5.31. Found: C, 63.80; H, 5.38; N, 5.27.

*Compound* **4bg**. Pale yellow oil. IR (cm^−1^, neat): 1072, 1173, 1481, 1615, 1721, 2978. ^1^H-NMR (CDCl_3_, 400 MHz) δ: 1.46 (t, 3H, *J* = 7.3 Hz), 2.64 (s, 3H), 4.52 (q, 2H, *J* = 7.3 Hz), 7.60 (dd, 1H, *J* = 9.4, 2.6 Hz), 7.20 (d, 1H, *J* = 2.6 Hz), 7.91 (s, 1H), 8.09 (d, 1H, *J* = 8.6 Hz). ^13^C-NMR (CDCl_3_, 100 MHz) δ: 14.5, 20.0, 62.3, 125.6, 129.6, 130.6, 131.3, 131.7, 134.2, 137.1, 144.4, 150.4, 166.6. GC-MS (70 eV): *m*/*z*: 249 ([M^+^], 9), 220 (18), 179 (31), 178 (15), 177 (100), 176 (18), 175 (18), 141 (24), 140 (40). Anal. Calcd. for C_13_H_12_ClNO_2_ (249.69): C, 62.53; H, 4.84; N, 5.61. Found: C, 62.50; H, 4.81; N, 5.58.

*Compound* **4ca**. Pale brown oil. IR (cm^−1^, neat): 1057, 1126, 1435, 1633, 1730, 2977. ^1^H-NMR (CDCl_3_, 400 MHz) δ: 1.36 (t, 3H, *J* = 7.3 Hz), 1.47 (t, 3H, *J* = 7.3 Hz), 3.04 (q, 2H, *J* = 7.3 Hz), 4.54 (q, 2H, *J* = 7.3 Hz), 7.74 (d, 1H, *J* = 8.9 Hz), 7.92 (d, 1H, *J* = 8.9 Hz), 8.11 (s, 1H), 8.49 (s, 1H). ^13^C-NMR (CDCl_3_, 100MHz) δ: 14.4, 15.0, 25.8, 29.8, 32.9, 62.3, 121.3, 123.6, 123.7, 126.7, 127.8, 127.9, 128.0, 128.1, 128.4, 130.4, 131.0, 131.7, 136.0, 137.7, 144.9, 152.3, 166.6. GC-MS (70 eV): *m*/*z*: 297 ([M^+^], 21), 268 (82), 225 (36), 224 (100), 223 (66), 222 (96), 197 (21), 196 (14), 154 (31). Anal. Calcd. for C_15_H_14_F_3_NO_2_ (297.28): C, 60.61; H, 4.75; N, 4.71. Found: C, 60.57; H, 4.78; N, 4.68.

*Compound* **4dh**. Yellow oil. IR (cm^−1^, neat): 1052, 1119, 1431, 1606, 1638, 1728, 2971. ^1^H-NMR (CDCl_3_, 400 MHz) δ: 1.46 (t, 3H, *J* = 7.3 Hz), 2.40–2.48 (m, 2H), 2.53 (s, 3H), 3.05-3.11 (m, 2H), 4.52 (q, 2H, *J* = 7.3 Hz), 4.97–5.07 (m, 2H), 5.80–5.93 (m, 1H), 7.50–7.55 (m, 2H), 7.94 (s, 1H), 8.09 (d, 1H, *J* = 9.4 Hz). ^13^C-NMR (CDCl_3_, 100 MHz) δ: 14.5, 22.0, 32.4, 35.3, 62.3, 115.9, 126.0, 129.1, 129.5, 132.2, 133.7, 137.2, 137.6, 138.6, 144.5, 149.2, 166.9. GC-MS (70 eV): *m*/*z*: 264 (7), 235 (14), 197 (37), 193 (100), 191 (50), 177 (11), 142 (11), 30 (10). Anal. Calcd. for C_17_H_19_NO_2_ (269.34): C, 75.81; H, 7.11; N, 5.20. Found: C, 75.77; H, 7.08; N, 5.22.

*Compound* **4di**. Yellow oil. IR (cm^−1^, neat): 697, 825, 1076, 1170, 1286, 1455, 1492, 1626, 1724, 2967. ^1^H-NMR (CDCl_3_, 400 MHz) δ: 1.25 (t, 3H, *J* = 7.3 Hz), 2.53 (s, 3H), 2.96 (t, 2H, *J* = 7.3 Hz), 5.51 (s, 2H), 7.31–7.42 (m, 3H), 7.48–7.56 (m, 4H), 7.94 (s, 1H), 8.07 (d, 1H, *J* = 8.5 Hz). ^13^C-NMR (CDCl_3_, 100 MHz) δ: 15.4, 22.0, 25.9, 67.8, 126.0, 128.6, 128.8, 128.9, 129.3, 129.6, 132.1, 135.8, 135.9, 136.0, 138.5, 144.5, 149.2, 166.9. GC-MS (70 eV): *m*/*z*: 210 (24), 196 (18), 168 (100), 167 (75), 165 (31), 152 (19), 141 (18), 114 (14), 90 (73), 65 (28). Anal. Calcd. for C_20_H_19_NO_2_ (305.38): C, 78.66; H, 6.27; N, 4.59. Found: C, 78.69; H, 6.24; N, 4.62.

## Figures and Tables

**Table 1:**
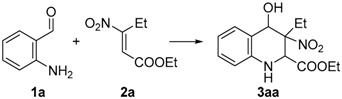
Optimization studies concerning the “*aza*-Michael-Henry domino process”.

Entry	1a (mmol)	2a (mmol)	Solvent	Temp. (°C)	Time (h)	Yield (%) ^1^
Entry 1	1	1	-	r.t.	24	Traces
Entry 2	1	1	-	50	24	44
Entry 3	1	1	-	60	24	51
Entry 4	1	1	-	70	18	61
Entry 5	1	1	-	80	18	52
Entry 6	1.1	1	-	70	18	59
Entry 7	1	1.1	-	70	18	66
Entry 8	1	1.2	-	70	18	64
Entry 9	1	1.1	MeCN	70	18	11
Entry 10	1	1.1	EtOAc	70	18	9
Entry 11	1	1.1	-	70	18	21 ^2^
Entry 12	1	1.1	-	70	18	8 ^3^

^1^ Yield of pure isolated product; ^2^ The reaction was performed in the presence of 1 eq. of TMG; ^3^ The reaction was performed in the presence of 1 eq. of Et_3_N.

**Table 2:**
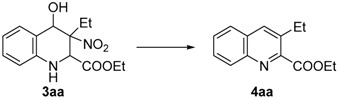
Optimization studies concerning aromatization step.

Entry	Base (eq.)	Solvent	Temp. (°C)	Time (h)	Yield (%) ^1^
Entry 1	TMG (1)	MeCN	50	24	47
Entry 2	DBU (1)	MeCN	50	24	36
Entry 3	Amberlyst A21 (1)	MeCN	50	24	Traces
Entry 4	TBD on polymer (1)	MeCN	50	24	62
Entry 5	Carbonate on polymer (1)	MeCN	50	24	57
Entry 6	BEMP on polymer (1)	MeCN	50	24	79
Entry 7	BEMP on polymer (0.8)	MeCN	50	24	67
Entry 8	BEMP on polymer (1.25)	MeCN	50	24	86
Entry 9	BEMP on polymer (1.5)	MeCN	50	24	82
Entry 10	BEMP on polymer (1.25)	MeCN	40	24	69
Entry 11	BEMP on polymer (1.25)	MeCN	60	24	85
Entry 12	BEMP on polymer (1.25)	EtOAc	50	24	71
Entry 13	BEMP on polymer (1.25)	2-MeTHF	50	24	47
Entry 14	BEMP on polymer (1.25)	Toluene	50	24	39

^1^ Yield of pure isolated product.

## Abbreviations

The following abbreviations are used in this manuscript:

TMG: 1,1,3,3-Tetramethylguanidine
DBU: 1,5-Diazabiciclo[5.4.0]undec-5-ene
BEMP: 2-*tert*-Butylimino-2-diethylamino-1,3-dimethylperhydro-1,3,2-diazaphosphorine
TBD: 1,5,7-Triazabicyclo[4.4.0]dec-5-ene
